# 

**Published:** 1897-09

**Authors:** 


					﻿CONTENTS.
ORIGINAL ARTICLES:
Successful Treatment of Sarcoma by Electrolysis and Cataphor-
esis Combined With the Internal use of Donovan’s Solution....	423
SOCIETY REPORTS:
Atlanta Society of Medicine............................. 432
SELECTIONS AND ABSTRACTS:
Chronic Specific Urethritis............................. 438
The Pathology of Recurrent Appendicitis................. 443
Inflammation of the Skin as a Result of the Poisonous Principles
of Plants...........................................   448
Is Malaria a Water-Borne Disease?....................... 448
Remedies That Enslave: How to Use Them Rightly.......... 458
Doctors of Osteopathy......................'............ 452
Suprapubic Cystotomy for Stone—Some Complications....... 454
The Danger of the Vaginal Injection .................... 457
SELECTIONS AND ABSTRACTS:
Five Successful Cases of General Suppurative Peritonitis Treated
by a New Method................................... 459
Pathology of Diabetes Insipidus.................. 464
Demonstration of the Pfeiffer-Widal Typhoid-Serum Reaction.. 465
EDITORIAL:
The Role of the Bacillus of Yellow Fever ........ 468
BUSINESS DEPARTMENT.........'........................ 470
PRESCRIPTION DEPARTMENT.............................. 475
				

